# A partial response to atezolizumab in alveolar soft part sarcoma with near-complete resolution of extensive pulmonary metastases: a case report

**DOI:** 10.3389/fonc.2026.1871862

**Published:** 2026-09-04

**Authors:** Joshua Ron Onichino, Dev Patel, Tom Powell, James Man Git Tsui, Ramy Saleh, Anthony Bozzo

**Affiliations:** 1 Faculty of Medicine and Health Sciences, McGill University, Montreal, QC, Canada; 2 Department of Diagnostic Radiology, McGill University Health Center, Montreal, QC, Canada; 3 Department of Oncology, Division of Radiation Oncology, McGill University Health Center, Montreal, QC, Canada; 4 Department of Oncology, McGill University Health Center, Montreal, QC, Canada; 5 Department of Orthopedic Surgery, McGill University Health Center, Montreal, QC, Canada

**Keywords:** alveolar soft part sarcoma, atezolizumab, immune checkpoint inhibitors, immunotherapy, PD-L1 inhibitors, pulmonary metastases, soft tissue sarcoma

## Abstract

Alveolar soft part sarcoma (ASPS) is a rare soft tissue sarcoma with high metastatic potential and limited response to systemic therapy. Biomarkers that predict which patients may benefit from emerging immune checkpoint inhibitors (ICIs) continue to surface. We report a case of stage IV ASPS with extensive pulmonary metastases and high PD-L1 expression that exhibited a marked response to atezolizumab, a PD-L1 antagonist. A 35-year-old man presented to an outside institution in October 2023 with a painless, progressively enlarging left thigh mass. Magnetic resonance imaging (MRI) demonstrated a 7.5 × 4.5 × 14 cm heterogeneous intramuscular lesion. Chest computed tomography (CT) revealed innumerable bilateral pulmonary metastases. Transthoracic biopsy of a pulmonary nodule confirmed stage IV ASPS with strong nuclear TFE3 positivity, Cathepsin K positivity, retained INI1 expression, and PD-L1 expression in 90-100% of malignant cells. The primary lesion was not characterized separately. Following transfer to our institution, atezolizumab was initiated in December 2023. At 3 months, chest CT demonstrated a marked regression of pulmonary metastases. Many resolved completely while others showed significant interval decrease in size. At 8 months, chest CT demonstrated further regression in size, number, and density of the pulmonary metastases. Only micronodules remained. At 12 months, chest CT demonstrated that all remaining pulmonary micronodules were stable without new or progressive disease. The primary lesion was treated with neoadjuvant hypofractionated radiotherapy and wide local resection. Post-operative histopathology of the primary lesion demonstrated >99% tumor necrosis with negative margins. This patient achieved a partial response of the primary thigh mass per Response Evaluation Criteria in Solid Tumors (RECIST) 1.1 and an immune partial response per immune RECIST (iRECIST). Moreover, this patient experienced a near-complete resolution of pulmonary metastatic disease sustained for 27 months despite an extensive metastatic burden. This case illustrates that substantial disease burden does not preclude meaningful, durable response to ICIs. Moreover, it raises the possibility that high PD-L1 expression may contribute to immune evasion. However, this observation was based on a metastatic biopsy. Prospective acquisition of standardized PD-L1 assay data, fusion-variant genotyping, and tumor-infiltrating lymphocyte profiles in future cases could inform individualized prognostication.

## 1 Introduction

Alveolar soft part sarcoma (ASPS) is an ultra-rare soft tissue sarcoma (STS) (1) accounting for less than 1% of all STS cases (2). It presents more commonly in adolescents and young adults between ages 15 and 39 years, with a slight female predominance (3). The typical primary tumor sites are the lower extremity in adults and the head and neck region in children (4). Molecularly, ASPS is characterized by the ASPSCR1::TFE3 fusion gene resulting from the der(17)t(X;17)(p11;q25) unbalanced translocation (5). This fusion gene drives tumor growth by activating transcriptional programs that regulate proliferation, angiogenesis, mitochondrial biogenesis, and differentiation (6). Microscopically, ASPS is characterized by uniform eosinophilic polygonal cells arranged in nest-like clusters separated by fibrovascular septa with periodic acid Schiff-positive and diastase-resistant intracytoplasmic crystals (7).

Despite its indolent growth pattern, ASPS demonstrates high metastatic potential, commonly spreading to the lung, bone, and brain (3). The median overall survival for patients presenting with stage IV disease is estimated to be 40 months with a 5-year survival rate of 20% (2). Moreover, in contrast to many other types of STS, ASPS is resistant to traditional anthracycline-based chemotherapy (3). Targeted therapies, including vascular endothelial growth factor (VEGF) receptor tyrosine kinase inhibitors (TKIs) and immune checkpoint inhibitors (ICIs), have demonstrated clinically meaningful activity (3, 8, 9). However, reported objective response rates (ORR) and progression-free survival (PFS) vary substantially across retrospective series and prospective trials, reflecting heterogeneous treatment response (3). Therefore, there is a critical need to identify clinical and biological factors that predict therapeutic benefit, independent of metastatic burden, in order to better select patients most likely to achieve durable response.

In this report, we present a patient with stage IV ASPS who presented with innumerable bilateral pulmonary metastases and strong programmed death-ligand 1 (PD-L1) expression at diagnosis. Following induction therapy with atezolizumab, a PD-L1 ICI, our patient achieved a partial response (PR) of the primary left thigh mass per Response Evaluation Criteria in Solid Tumors (RECIST) 1.1 and an immune partial response (iPR) per immune RECIST (iRECIST), with near-complete resolution of pulmonary metastatic disease. Most pulmonary metastases resolved completely, and the remaining lesions regressed to micronodular residuals without evidence of new metastatic disease.

## 2 Case description

### 2.1 Patient information

A 35-year-old man presented to an outside institution in October 2023 with a painless, progressively enlarging left thigh mass. Aside from the thigh mass, the patient was clinically healthy. His history was notable for 15 years of traditional cigarette smoking followed by 5 years of electronic cigarette smoking.

### 2.2 Clinical findings

Physical examination demonstrated a palpable left thigh mass without overlying skin changes or inguinal lymphadenopathy. The left lower extremity was neurovascularly intact. An occasional cough was observed. There was no evidence of weight loss.

### 2.3 Diagnostic assessment

**Figure 1:**
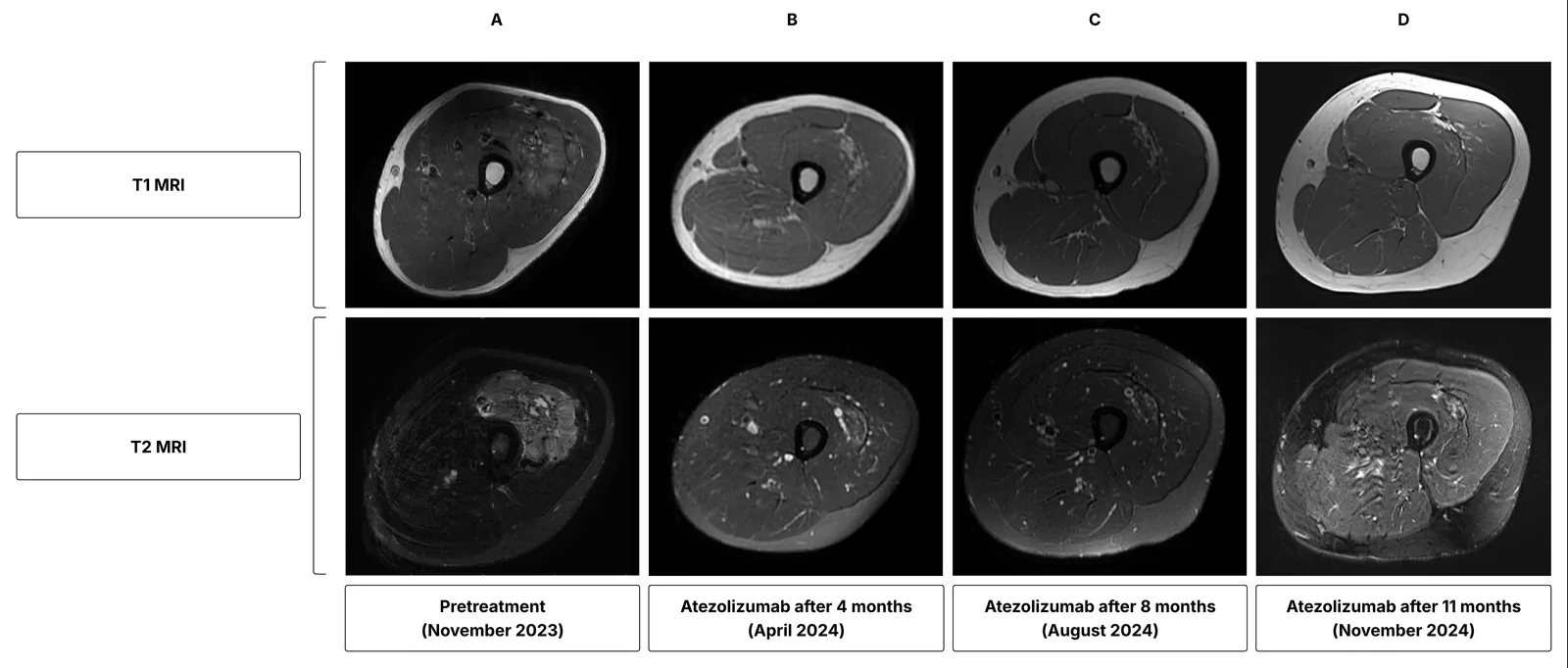
Axial MRI of the left thigh before and during treatment. **(A)** Pre-treatment MRI obtained at an outside institution in November 2023 demonstrating a large heterogeneous intramuscular left thigh mass. **(B)** MRI obtained in April 2024, 4 months after initiation of atezolizumab, demonstrating interval decrease in size and heterogeneity of the mass. **(C)** MRI obtained in August 2024, 8 months after initiation of atezolizumab, demonstrating further, more dramatic reduction in lesion size. **(D)** MRI obtained in November 2024, 11 months after initiation of atezolizumab, following completion of neoadjuvant radiotherapy and prior to surgical resection, demonstrating further reduction in lesion size. T1- and T2-weighted sequences are shown for each timepoint. MRI, magnetic resonance imaging.

**Figure 2:**
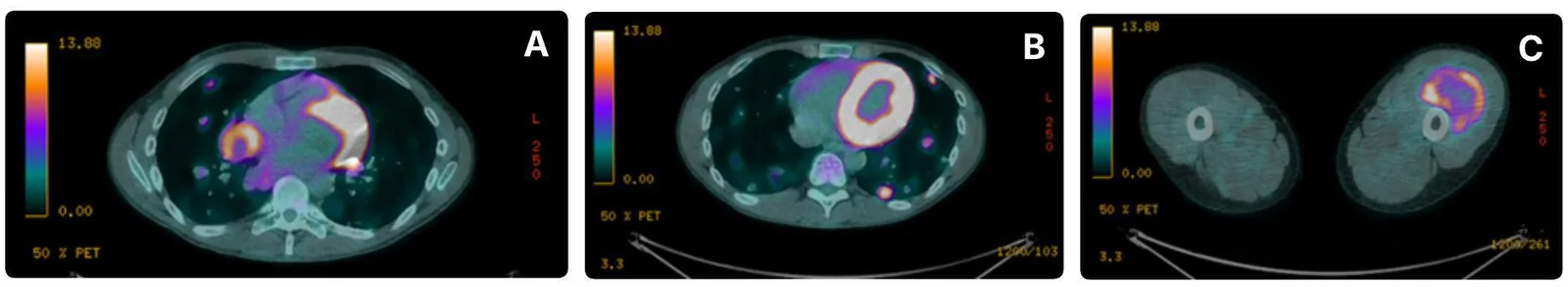
Baseline PET-CT demonstrating primary and metastatic disease. Baseline PET-CT demonstrating metabolically active primary and metastatic disease. **(A, B)** Axial PET-CT images through the chest obtained at diagnosis, demonstrating metabolically active thoracic metastatic disease in the setting of extensive bilateral pulmonary metastases. **(C)** Axial PET-CT image through the thighs demonstrating intense metabolic activity within the primary left thigh soft tissue mass. PET-CT, positron emission tomography-computed tomography.

**Figure 3:**
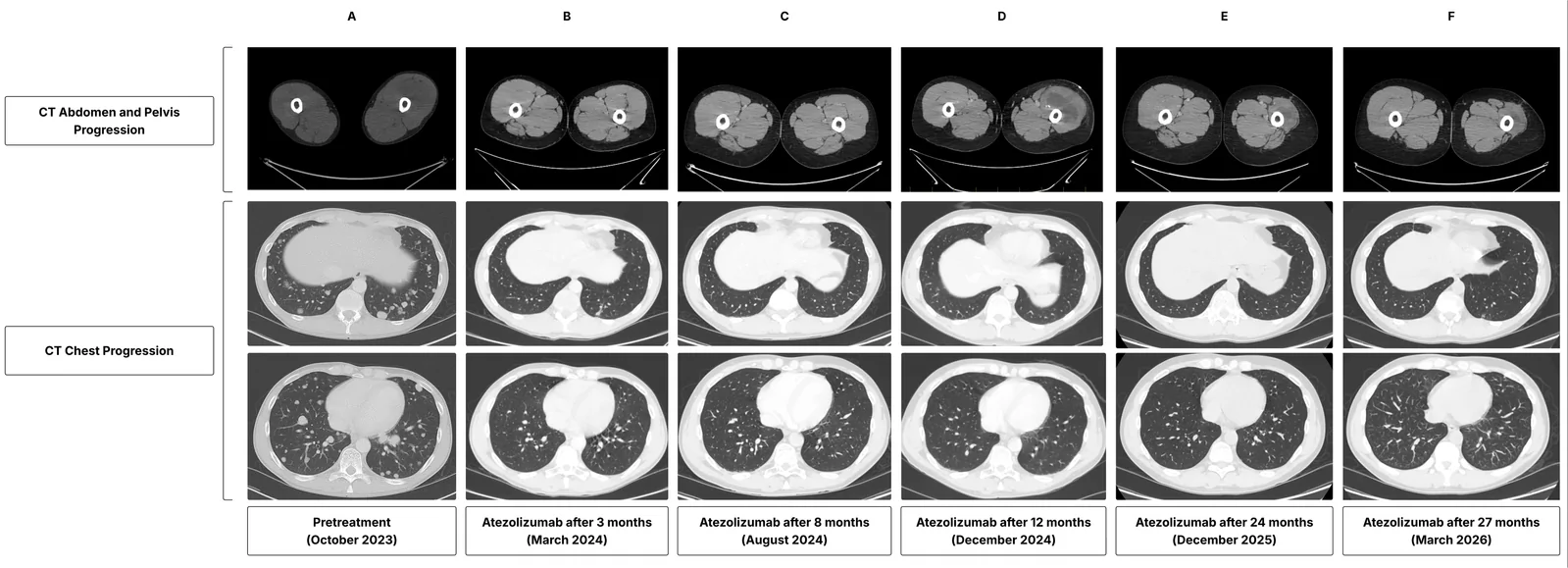
CT imaging demonstrating treatment response over time. Axial CT images demonstrating atezolizumab treatment response over time across the primary and metastatic lesions. The top row shows abdomen and pelvis CT at the level of the thighs that was obtained to monitor the primary lesion and screen for extrapulmonary and extrathoracic disease. The middle and bottom rows show chest CT demonstrating reduction of pulmonary metastatic disease. **(A)** Pre-treatment baseline imaging obtained at an outside institution in October 2023, demonstrating innumerable bilateral pulmonary metastases. **(B)** Chest CT in March 2024, 3 months after initiation of atezolizumab, demonstrating marked interval reduction in the number and size of pulmonary metastases. Almost all nodules completely resolved, and only a few millimetric micronodules remained. **(C)** Chest CT in August 2024, 8 months after initiation of atezolizumab, demonstrating further regression of residual micronodules. **(D)** Chest CT in December 2024, 12 months after initiation of atezolizumab, demonstrating stable residual micronodules without evidence of progression or new lesions. **(E)** Chest CT in December 2025, 24 months after initiation of atezolizumab, demonstrating continued absence of measurable pulmonary metastatic disease. **(F)** Chest CT in March 2026, 27 months after initiation of atezolizumab, demonstrating sustained near-complete response. Throughout follow-up, abdomen and pelvis CT showed no evidence of extrapulmonary or extrathoracic metastatic disease. CT, computed tomography.

Diagnostic assessment was performed at an outside institution in October and November 2023. It included magnetic resonance imaging (MRI) of the left thigh, obtained in November 2023, which demonstrated a heterogeneous intramuscular mass measuring approximately 7.5 × 4.5 × 14 cm and wrapping around roughly 180° of the left femur (**Figure 1A**). Positron emission tomography-computed tomography (PET-CT) demonstrated that the left thigh lesion was metabolically active, with uptake in the primary lesion as well as metabolically active foci (**Figure 2**). Staging chest CT revealed innumerable bilateral pulmonary metastases, with the largest lesion measuring 3 cm, consistent with stage IV disease (**Figure 3A**). Transthoracic biopsy of a left lower lobe pulmonary nodule and subsequent histopathologic assessment was performed. Immunohistochemical analysis of the lung nodule showed strong nuclear TFE3 positivity, Cathepsin K positivity, retained INI1 expression, and PD-L1 expression in 90-100% of malignant cells. The specific PD-L1 antibody clone, staining platform, scoring system, and positivity criteria were not specified. Molecular confirmation of the ASPSCR1::TFE3 fusion was not performed, and the fusion variant (type 1 versus type 2) was therefore not determined. The diagnosis of ASPS was made based on the characteristic histologic appearance of the biopsied tissue along with the immunohistochemical findings described above. Biopsy and immunohistochemical analysis of the primary lesion were not performed.

### 2.4 Therapeutic intervention

Atezolizumab was initiated in December 2023. It was administered as a fixed intravenous dose of 1200 mg in 250 mL normal saline infused over 60 minutes for the first cycle and 30 minutes for all subsequent cycles. Infusions were to be administered every 3 weeks, resulting in 21 days per treatment cycle. No fixed total number of cycles was planned as treatment was intended to continue indefinitely until disease progression or unacceptable toxicity, with ongoing reassessment throughout the patient’s 10-year surveillance period. Following multidisciplinary review in September 2024, a local consolidation strategy was planned for the primary thigh lesion consisting of neoadjuvant hypofractionated radiation therapy followed by surgical resection. Neoadjuvant radiotherapy was delivered from October 2024 to November 2024. The patient was treated with photon-based radiotherapy using 6 MV photons, delivered via intensity-modulated radiotherapy (IMRT) using the RapidArc technique. The prescribed dose was 30 Gy in 5 fractions, administered every other day. For simulation, the patient was positioned feet-first and supine, with the arms placed across the chest, and immobilized using a Vac-Lok device. CT and MRI simulation images were acquired and co-registered for target delineation. The gross tumor volume (GTV) was defined based on residual disease visible on the co-registered CT and MRI simulation scans. A 1.5 cm radial expansion and a 3 cm superior-inferior expansion were applied to generate the clinical target volume (CTV), which was subsequently cropped to respect anatomical boundaries (e.g., bone). In addition, the pre-systemic therapy diagnostic MRI was registered to the simulation images, and the pre-treatment GTV (including edema) was combined with the CTV. A 0.7 cm isotropic expansion was then applied to generate the planning target volume (PTV). The patient completed radiotherapy without any treatment-related toxicity. Wide local resection of the left thigh mass was subsequently performed in December 2024. Throughout treatment, multidisciplinary discussion involving radiation oncology, medical oncology, radiology, pathology, orthopedic surgery, and surgical oncology guided ongoing management and surveillance for disease progression while on atezolizumab.

### 2.5 Follow-up and outcomes

Follow-up chest CT performed in March 2024, 3 months after initiation of atezolizumab, demonstrated near-complete PR and iPR by RECIST 1.1 and iRECIST, respectively. For a response to be classified as a PR, up to 5 target lesions in total and no more than 2 per organ are selected at baseline. A patient experiences a PR if there is at least a 30% decrease in the sum of target lesion diameters relative to baseline in the absence of new lesions (10). iRECIST retains similar measurement principles to RECIST 1.1 and was developed for immunotherapy trials to better account for atypical immune-related response patterns (11).

Most pulmonary metastases resolved completely, and the remaining lesions regressed to micronodular residuals, with no evidence of new metastatic disease (**Figure 3B**). Follow-up chest CT in August 2024, 8 months after initiation of atezolizumab, demonstrated further regression in the size, number, and density of the bilateral metastatic pulmonary nodules (**Figure 3C**). Subsequent chest CT in December 2024, 12 months after initiation, demonstrated residual pulmonary micronodules that remained stable compared to prior imaging, with no evidence of progression or new lesions (**Figure 3D**). Follow-up chest CT in December 2025, 24 months after initiation, demonstrated continued absence of measurable pulmonary metastatic disease, with residual pulmonary micronodules stable in size and number compared to prior imaging (**Figure 3E**). These residual micronodules did not meet criteria for an oligometastatic or oligoresidual state, as they were considered to represent no evidence of active disease rather than a distinct residual burden. Concurrent abdomen and pelvis CT at each timepoint demonstrated no evidence of extrapulmonary or extrathoracic metastatic disease and progressive interval decrease in the size of the primary thigh lesion (**Figure 3**). These findings were consistent across MRI of the primary thigh lesion, as well (**Figure 1**).

**Figure 4:**
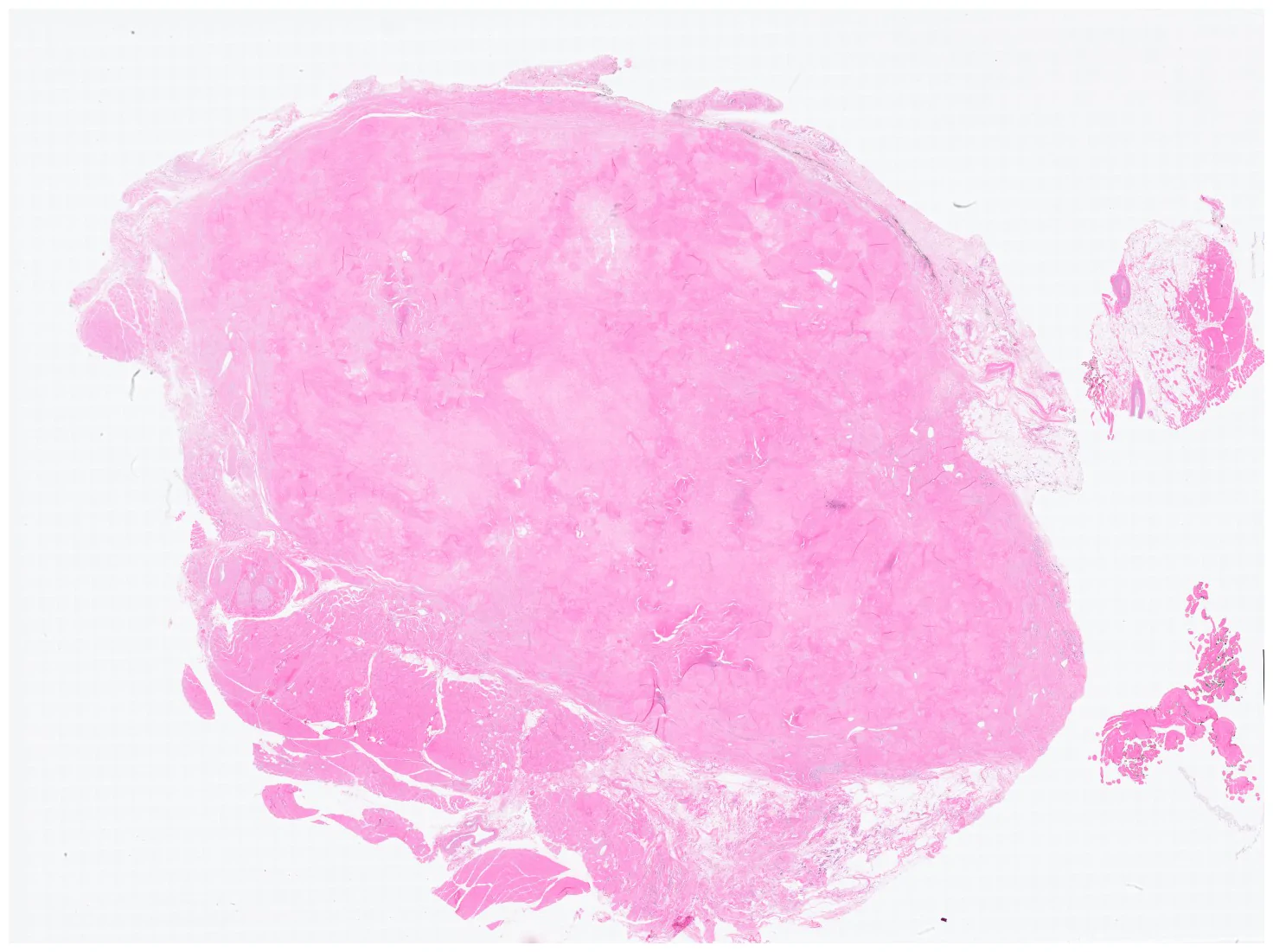
Post-operative histopathology of the primary lesion. Post-operative histopathology of the resected left thigh mass. H&E-stained section demonstrating >99% tumor necrosis without viable malignant cells, consistent with a near-complete pathologic response. All surgical margins were uninvolved by sarcoma, and lymphovascular invasion was absent. Immunohistochemical staining was not performed on this specimen. H&E, hematoxylin and eosin.

Prior to the initiation of neoadjuvant radiotherapy in October 2024, MRI of the left thigh mass obtained in April 2024 and August 2024, 4 and 8 months after initiation of atezolizumab, respectively, showed a dramatic reduction in the size of the primary lesion (**Figures 1B, C**). These findings suggest that the primary tumor regressed in response to atezolizumab prior to the induction neoadjuvant radiotherapy. Further MRI of the left thigh mass in November 2024, 11 months after initiation of atezolizumab, performed after neoadjuvant radiotherapy showed a further reduction in the size of the primary lesion (**Figure 1D**). After neoadjuvant radiotherapy and wide local excision in December 2024, post-operative histopathology demonstrated >99% tumor necrosis (**Figure 4**). Given that resection followed both atezolizumab and neoadjuvant radiotherapy, the relative contribution of each treatment modality to this near-complete pathologic response cannot be determined. All surgical margins were uninvolved by sarcoma, with the closest margin measuring 0.3 cm anteriorly and all other margins measuring >1.0 cm. Immunohistochemical analysis was not performed on the resected specimen. The proximal vessel margin biopsy was negative, and lymphovascular invasion was absent. The post-operative course was uncomplicated, and the patient was subsequently placed on surveillance according to STS standards, with planned long-term follow-up for 10 years.

The patient has continued maintenance atezolizumab in the post-operative period. In July 2024, he developed immune-related colitis, graded as Common Terminology Criteria for Adverse Events (CTCAE) v5.0 Grade 3 which was initially managed with prednisone alone. Atezolizumab infusions were intermittently interrupted over the subsequent months due to recurrent colitis and infusions were fully discontinued in June 2025. Infliximab was subsequently added for ongoing management of the colitis, with a second course, to be administered every 8 weeks, given in December 2025. Immune-related gynecomastoid hyperplasia, graded as CTCAE v5.0 Grade 2 and confirmed by core needle biopsy in June 2025, was identified separately and did not contribute to the treatment interruption. Atezolizumab was resumed on a consistent 3-week infusion schedule in January 2026 and has continued without further interruption. As of March 2026, the patient has completed 27 cycles of atezolizumab. Routine surveillance chest CT demonstrates sustained near-complete response. Since the dramatic initial reduction in his diffuse bilateral metastatic disease – diagnosed prior to transfer in late 2023 – and near-complete PR by March 2024 zero nodules have progressed and no new nodules have been reported. This sustained response is current through his most recent chest CT from March 2026 (**Figure 3F**), resulting in a PFS of 27 months.

Baseline laboratory evaluation was notable only for leukocytosis (12.0 ×10^9^/L) with neutrophilia (10.5 ×10^9^/L). Renal function, electrolytes, liver enzymes, and thyroid function were within normal limits. At the time atezolizumab was held for treatment-related colitis in June 2025, white cell and neutrophil counts had normalized (9.7 and 6.3 ×10^9^/L, respectively). Renal, hepatic, and electrolyte parameters were stable. Following resumption of atezolizumab in January 2026, laboratory parameters remained stable, with no new hematologic, renal, hepatic, or metabolic abnormalities identified.

**Table 1** summarizes the details of the case presented in this report.

**Table 1:** Timeline and the applied clinical interventions.

Time point	Episode of care	Key data
October-November 2023	Presentation & Diagnostic Workup	The patient presented with a painless, progressively enlarging left thigh mass. Pre-treatment MRI showed a heterogeneous intramuscular left thigh mass measuring 7.5 × 4.5 × 14 cm. Chest CT showed innumerable bilateral pulmonary metastases. Transthoracic biopsy of a pulmonary nodule in the left lower lobe confirmed metastatic ASPS with PD-L1 expression in 90-100% of malignant cells.
December 2023	Systemic Therapy Initiation	The patient was transferred to our institution and atezolizumab was initiated.
March-April 2024	Early Response Assessment	Follow-up chest CT demonstrated near-complete resolution of measurable pulmonary disease. Remaining lesions regressed to micronodular residuals. There was no evidence of new metastatic disease. MRI showed interval decrease in size of the primary left thigh lesion.
July 2024	Treatment-Related Adverse Event	On follow-up, the patient developed immune-related colitis graded as CTCAE v5.0 Grade 3 that was initially managed with prednisone alone.
August 2024	Further Response Assessment	MRI showed a further, more dramatic reduction in the size of the primary left thigh lesion. Chest CT demonstrated further regression in size, number, and density of the pulmonary metastases.
September 2024	Local Consolidation Planning	Multidisciplinary planning was initiated for neoadjuvant radiation followed by wide resection of the primary lesion.
October-November 2024	Neoadjuvant Radiotherapy	The regimen involved photon-based IMRT (RapidArc technique, 6 MV), 30 Gy in 5 fractions every other day. The radiotherapy was completed without treatment-related toxicity.
November 2024	Post-Radiotherapy Response Assessment	MRI showed further reduction in the size of the primary lesion prior to resection.
December 2024	Surgical Resection & Pathology	The patient underwent wide local resection of the primary lesion. Histopathology showed >99% tumor necrosis with negative margins, consistent with a near-complete pathologic response.
June 2025	Atezolizumab Discontinued & Gynecomastoid Hyperplasia Identified	Atezolizumab was fully discontinued following recurrent immune-related colitis graded as CTCAE v5.0 Grade 3. Infliximab was initiated for ongoing management of the colitis. Immune-related gynecomastoid hyperplasia graded as CTCAE v5.0 Grade 2 was identified separately and confirmed by core needle biopsy. This finding did not contribute to the hold.
December 2025	Further Response Assessment & Infliximab Course	Abdomen and pelvis CT continued to demonstrate no evidence of extrapulmonary or extrathoracic metastatic disease. Chest CT continued to demonstrate absence of measurable pulmonary disease. A second course of infliximab (administered every 8 weeks) was given for ongoing colitis management.
January 2026	Atezolizumab Resumed	Atezolizumab was resumed on the previous schedule.
March 2026	Surveillance Follow-up	Surveillance chest CT demonstrated a maintained near-complete PR and iPR. The patient remains progression-free at 27 months and counting.

ASPS, alveolar soft part sarcoma; CT, computed tomography; CTCAE, Common Terminology Criteria for Adverse Events; Gy, gray; IMRT, intensity-modulated radiotherapy; iPR, immune partial response; PD-L1, programmed death-ligand 1; PR, partial response; MRI, magnetic resonance imaging.

## 3 Discussion

We presented a rare case of stage IV ASPS who achieved a PR and an iPR following atezolizumab induction despite presenting with innumerable bilateral pulmonary metastases. Transthoracic biopsy of lung metastases revealed PD-L1 expression on 90-100% of malignant cells. This case illustrates that substantial metastatic burden does not preclude meaningful response to ICIs. It also raises the hypothesis that high PD-L1 expression may be associated with response to ICI in ASPS. However, this observation was based on a metastatic biopsy.

Despite recent advances in ICIs, stage IV ASPS remains therapeutically challenging. ASPS follows an indolent clinical course that delays diagnosis and contributes to a high rate of metastatic disease at presentation (12). Consequently, many patients are treated in the setting of advanced disease, where durable responses remain difficult to predict.

The current management of stage IV ASPS centers on systemic therapy with VEGF receptor TKIs, ICIs, or combination therapies (3). In a phase II study of atezolizumab involving 52 patients with advanced ASPS, an objective response was observed in 19 (37%) patients, including 18 PRs and one complete response (13). Responses occurred across the full range of baseline PD-L1 and PD-1 expression. Moreover, responses were associated with intratumoral dispersal of CD8+ T cells rather than with the proportion of PD-L1 positive tumor cells (13). The median PFS in this study was 20.8 months (13). In a separate phase II study of atezolizumab involving 20 patients with advanced ASPS, an objective response was observed in 2 (10%) patients, including 2 complete responses (14). The median PFS in this study was 10.0 months. While the ORR in this study (10%) was lower than that which was reported in Chen et al. (37%), Kojima et al. included two durable complete responses and identified greater pre-treatment infiltration of CD8+PD-1+ T cells, rather than PD-L1 expression, as the principal feature distinguishing responders (14). In this context, the magnitude and durability of the near-complete response (27 months) observed in our patient despite extensive metastatic burden compare favorably with published outcomes and exceed the reported median PFS.

Radiotherapy was not employed in the management of our patient’s lung metastases. This treatment modality may provide benefit for brain metastases (15) but is generally not performed for lung metastases (3). However, one case report presented beneficial effects of a hyperfractionated dose of 44.8 Gy administered to a patient with stage IV ASPS lung metastases (16).

Moreover, surgical excision of the primary lesion in stage IV ASPS remains controversial in the era of effective systemic therapy. While analyses from the Surveillance, Epidemiology, and End Results (SEER) and National Cancer Data Base (NCDB) databases in the United States of America suggest that surgical resection of the primary lesion is associated with improved overall survival, analyses from the Bone and Soft Tissue Registry (BSTTR) database in Japan did not (12, 17, 18). In our case, surgical resection of the primary lesion followed both atezolizumab and neoadjuvant radiotherapy. Post-operative histopathology demonstrated >99% tumor necrosis without viable malignant cells, indicating a near-complete pathological response (**Figure 4**). Notably, the marked regression of the left thigh mass demonstrated by the MRI performed in August 2024 (**Figure 1C**), prior to the induction of neoadjuvant radiotherapy, suggests a meaningful systemic effect on the primary tumor.

The marked PD-L1 expression observed in this case raises important questions regarding this patient’s ASPS microenvironment. Programmed cell death protein 1 (PD-1) is an inhibitory receptor expressed on activated T and B cells, while its ligand, PD-L1, is expressed on antigen-presenting cells and tumor-infiltrating lymphocytes but can also be expressed on tumor cells. Physiologic engagement of PD-1 by PD-L1 functions as an immune checkpoint that limits excessive immune responses by suppressing T cell activation and proliferation. ICIs such as atezolizumab inhibit PD-L1 thereby releasing this inhibitory signal and restoring CD8+ T cell activity against tumor cells (19).

In ASPS, PD-L1 expression within the tumor microenvironment may be influenced by oncogenic signaling pathways associated with the ASPSCR1::TFE3 fusion. TFE3 activity has been linked to activation of the transforming growth factor-β (TGF-β) pathway, which can promote an inflammatory tumor microenvironment that enhances PD-L1 expression (20, 21). TGF-β signaling also supports the differentiation and maintenance of regulatory T cells through induction of FOXP3 expression, further contributing to immune suppression (21). These mechanisms suggest that PD-L1 expression in this patient’s ASPS may have represented an adaptive strategy of immune evasion. Emerging data also suggest that the ASPSCR1::TFE3 fusion variant may shape the tumor immune microenvironment. Chen et al. found that tumors harboring the type 2 fusion exhibited a paucity of activated CD8+ tumor-infiltrating lymphocytes and a markedly lower ORR than type 1 tumors (0% versus 43.9%) (22).

Moreover, PD-L1 expression alone may not reliably predict response to ICIs. Clinical studies in ASPS, non-small cell lung cancer, melanoma, and renal cell carcinoma demonstrate that responses to ICIs can occur irrespective of tumor PD-L1 expression (20, 23). Indeed, Conley et al. presented a case of stage IV ASPS refractory to VEGF receptor TKIs with metastases to the lung, liver, pericardium, and omentum that eventually saw an iPR upon induction of ICI combination therapy with ipilimumab and nivolumab (20). However, PD-L1 expression in this patient’s tumor was negative (20), illustrating that PD-L1 expression is not required for response. One proposed explanation for ICI effectiveness in the absence of PD-L1 expression is that checkpoint inhibition can induce systemic immune activation. This activation leads to clonal expansion of CD8+ T cells and the generation of new tumor-reactive T cell populations in peripheral blood and tumor-draining lymph nodes that subsequently migrate to the tumor microenvironment and mediate antitumor activity (24).

These data suggest that the density and functional state of tumor-infiltrating lymphocytes, rather than PD-L1 expression alone, may more reliably predict response to atezolizumab in ASPS. Kojima et al. found that atezolizumab responders demonstrated significantly greater pre-treatment infiltration of CD8+PD-1+ T cells and lower expression of exhaustion markers Tim-3 and LAG-3. These observations led the investigators to propose CD8+PD-1+ T cell density as a candidate predictive biomarker of response to atezolizumab in ASPS (14). Consistent with this finding, Chen et al. observed responses irrespective of baseline PD-L1 expression, with response instead tracking with the intratumoral dispersal of CD8+ T cells (13). The high PD-L1 expression observed in our patient should therefore be interpreted within this broader context.

Interpretation of the PD-L1 result in this case is further limited by the absence of assay details. Among the validated PD-L1 immunohistochemistry assays, the SP142 clone used as the companion diagnostic for atezolizumab stains the fewest tumor cells and is the least sensitive. By contrast, the 22C3, 28-8, and SP263 assays are more concordant and the 73–10 assay is the most sensitive (25). Given that the antibody clone, platform, and scoring system used in our patient were not available, the reported 90-100% expression cannot be benchmarked against these assays, and cross-study comparison of PD-L1 positivity in ASPS should be made with caution.

In STS more broadly, PD-L1 expression varies considerably between histologies (26–28). Higher PD-L1 expression has been reported in epithelioid sarcoma and undifferentiated pleomorphic sarcoma, whereas lower expression has been reported in mesenchymal chondrosarcoma. Importantly, the prognostic and predictive value of PD-L1 expression remains inconsistent across STS studies (20, 26, 28).

Tumor mutational burden may also influence response to ICIs. High mutational burden has been associated with increased neoantigen load and improved outcomes following ICI therapy, suggesting that hypermutated tumors may derive greater benefit from these agents (19). However, translocation-associated sarcomas such as ASPS typically exhibit a low mutational burden (6, 29), indicating that mechanisms beyond neoantigen load may contribute to immunotherapy sensitivity in this disease. Consistent with this observation, meaningful responses to ICIs have also been reported in other sarcomas with relatively low or variable mutational burden but strong PD-L1 expression, including angiosarcoma and rhabdomyosarcoma (30, 31). However, isolated case reports cannot establish which immune feature drove the response.

These observations highlight the complex relationship between PD-L1 expression, tumor immunogenicity, and response to ICIs. While PD-L1 expression may enrich for responders in some contexts, its predictive value remains incomplete, and combination immunotherapy strategies have demonstrated survival benefit regardless of PD-L1 expression (13). In this context, the exceptionally high PD-L1 expression observed in our patient raises the hypothesis that PD-L1-mediated immune suppression contributed to tumor immune evasion and that its blockade may have played a role in the marked response observed. However, the contribution of PD-L1 status to the response observed in this case cannot be definitively established and should be interpreted as hypothesis-generating.

Beyond the PD-L1 hypothesis, the principal clinical significance of this case lies in the extent and durability of response achieved despite an unusually extensive metastatic disease burden. Innumerable bilateral pulmonary metastases at presentation are associated with substantially worse outcomes in ASPS. Metastatic disease at diagnosis is the strongest adverse prognostic factor identified in population-based cohorts with a 5-year disease-specific survival of 62% versus 86% for localized disease (12). Moreover, stage IV disease carries a median overall survival of only 40 months (2). A more recent cohort similarly reported a 5-year overall survival of 67.7% following metastatic diagnosis, with immunotherapy achieving the highest response rate (40%) among first-line systemic options, ahead of TKIs (26%) and chemotherapy (7%) (32). Against this backdrop, near-complete radiographic resolution sustained for 27 months represents a response substantially exceeding expectations for this disease burden.

## 4 Limitations

This case has several limitations. First, the primary thigh lesion was not biopsied or characterized immunohistochemically prior to treatment. The immunohistochemical profile described reflects pulmonary metastasis only and cannot be assumed to represent the primary tumor. However, biopsy of the primary lesion was obtained post-operatively and, despite not being characterized immunohistochemically, demonstrated >99% tumor necrosis with negative margins. Second, the antibody clone, staining platform, scoring system, and positivity criteria used for PD-L1 assessment were not available, precluding benchmarking against validated assays and limiting cross-study comparison. Third, molecular confirmation of the ASPSCR1::TFE3 fusion was not performed, so the fusion variant (type 1 versus type 2), which are increasingly recognized as biologically and immunologically distinct, remains unknown. Fourth, CD8+ and CD8+PD-1+ tumor-infiltrating lymphocyte density was not assessed, precluding evaluation of the biomarker that recent data (13, 14) suggest may be more predictive of atezolizumab response than PD-L1 expression itself. Finally, because surgical resection followed both atezolizumab and neoadjuvant radiotherapy, the relative contribution of each modality to the near-complete pathologic response cannot be determined.

Given the rarity of ASPS and the limited prospective data available, we encourage clinicians managing similar patients to prospectively collect biopsy material from both primary and metastatic sites, standardized PD-L1 assay data, fusion-variant genotyping, and tumor-infiltrating lymphocyte quantification wherever feasible, as these variables may ultimately prove more informative for prognosis and treatment selection than any single case can establish.

## 5 Patient perspective

Receiving this diagnosis was an overwhelming experience for this patient and his family. Just one week before learning he had cancer, he and his wife had found out that she was pregnant. What should have been a time of excitement quickly became one of fear and uncertainty. The knowledge that the disease had already spread to his lungs made the diagnosis especially difficult to process. As treatment progressed and the tumor responded, he gradually began to regain hope. Now, being here with his wife and child, he remains deeply grateful for the care he received and for the opportunity to continue moving forward with his family.

## Data Availability

The original contributions presented in the study are included in the article/supplementary material. Further inquiries can be directed to the corresponding author.
